# Area level measures of socioeconomic deprivation and their impact on very preterm birth and very preterm birth outcomes: A retrospective cohort study

**DOI:** 10.1038/s41372-026-02674-1

**Published:** 2026-04-20

**Authors:** Daniel O’Reilly, Lauren Murphy, Sharon Dempsey, Isobel Claire Gormley, Anna Curley, Carmel Maria Moore, Naomi McCallion

**Affiliations:** 1 Department of Neonatology, Rotunda Hospital, Parnell Square, Dublin, 1 Ireland; 2 Department of Neonatology, National Maternity Hospital, Holles Street, Dublin, 2 Ireland; 3 Department of Biomedical and Biomolecular Science, Conway Institute, University College Dublin, Belfield, Ireland; 4 School of Mathematics and Statistics, University College Dublin, Belfield, Ireland; 5 Department of Paediatrics, Royal College of Surgeons Ireland, St.Stephens Green, Dublin, 2 Ireland

**Keywords:** Epidemiology, Health policy

## Abstract

**Objective:**

We hypothesised that socioeconomic deprivation as determined by an area measure would be an important risk factor for preterm birth in a contemporary cohort.

**Methods:**

A retrospective study was carried out across two tertiary maternity hospitals in Ireland between 2018 and 2022 representing approximately 27% of all births in Ireland. Non stillborn infants whose address could be matched to a small area deprivation index (Haase-Pratschke (HP) Index) were included in the analysis. A combination of non-parametric and parametric tests were used to examine the impact of socioeconomic deprivation.

**Results:**

80,231 births were identified from 2018 to 2022. After exclusion criteria were applied, 912 very preterm births were available for analysis. There was a significant difference in population distributions of area level socioeconomic measures of neonates born <32 weeks and those born >=32 weeks(*p* < 0·001).

**Conclusion:**

Socioeconomic deprivation remains an important determinant of very preterm birth although has limited effect on acute neonatal outcomes.

## Introduction

Preterm birth, defined as being born less than 37 weeks corrected gestational age, is an important cause of morbidity and mortality. Globally, the majority of deaths in children under 5 occur in the neonatal period [1] and preterm neonates account for 40% of these deaths. Although very preterm neonates (<32 weeks corrected gestational age) are a small population they typically require high levels of support and are also at high risk of unique complications of prematurity such as intraventricular haemorrhage (IVH) and chronic lung disease/bronchopulmonary dysplasia (CLD/BPD). These conditions in turn are associated with a higher incidence of adverse neurodevelopmental outcomes [2].

Socioeconomic deprivation has been associated with preterm birth in a number of studies, predominantly in the United Kingdom and United States [3–5] but studies specifically relating to very preterm birth are limited. Studies to date have shown an increase in very preterm birth in families from areas of higher socioeconomic deprivation, but no differences in complications associated with preterm birth [3, 6, 7].

The Irish healthcare system has been described as unique amongst European countries. Whilst poverty has fallen in Ireland over recent decades, it remains the only European country without universal primary health care coverage [8, 9]. An area level deprivation index Haase-Pratschke (HP) index (https://data.pobal.ie/Portal/apps/sites/#/pobal-maps) has been previously utilised to examine the effect of relative socioeconomic status on Irish healthcare outcomes [10, 11]. This multifactorial index uses ten measures from Census 2022 including educational attainment, employment status and numbers living in individual households within a small area (SA) encompassing 50–200 households each [12]. Notably whilst deprivation overall improved nationally between editions of the HP index, the gap between most disadvantaged and average areas has increased [12].

We hypothesised that socioeconomic deprivation as determined by the HP Index would be an important risk factor for preterm birth in a contemporary, Irish cohort. Our secondary hypothesis was that neonates from areas with greater deprivation would experience more adverse outcomes associated with very preterm birth.

## Methods

### Study design

This was retrospective cohort study conducted across two large tertiary maternity hospitals from 2018 to 2022. All livebirths during this time period were identified on the Maternal Neonatal Clinical Management System (MN-CMS), an electronic health record which is shared across both sites. Ethical approval was granted on both sites by their research ethics committees (NMH REC EC28.2024/ Rotunda REC 2023-009) and all methods were performed in accordance with relevant guidelines and regulations.

## Participants

**Fig. 1:**
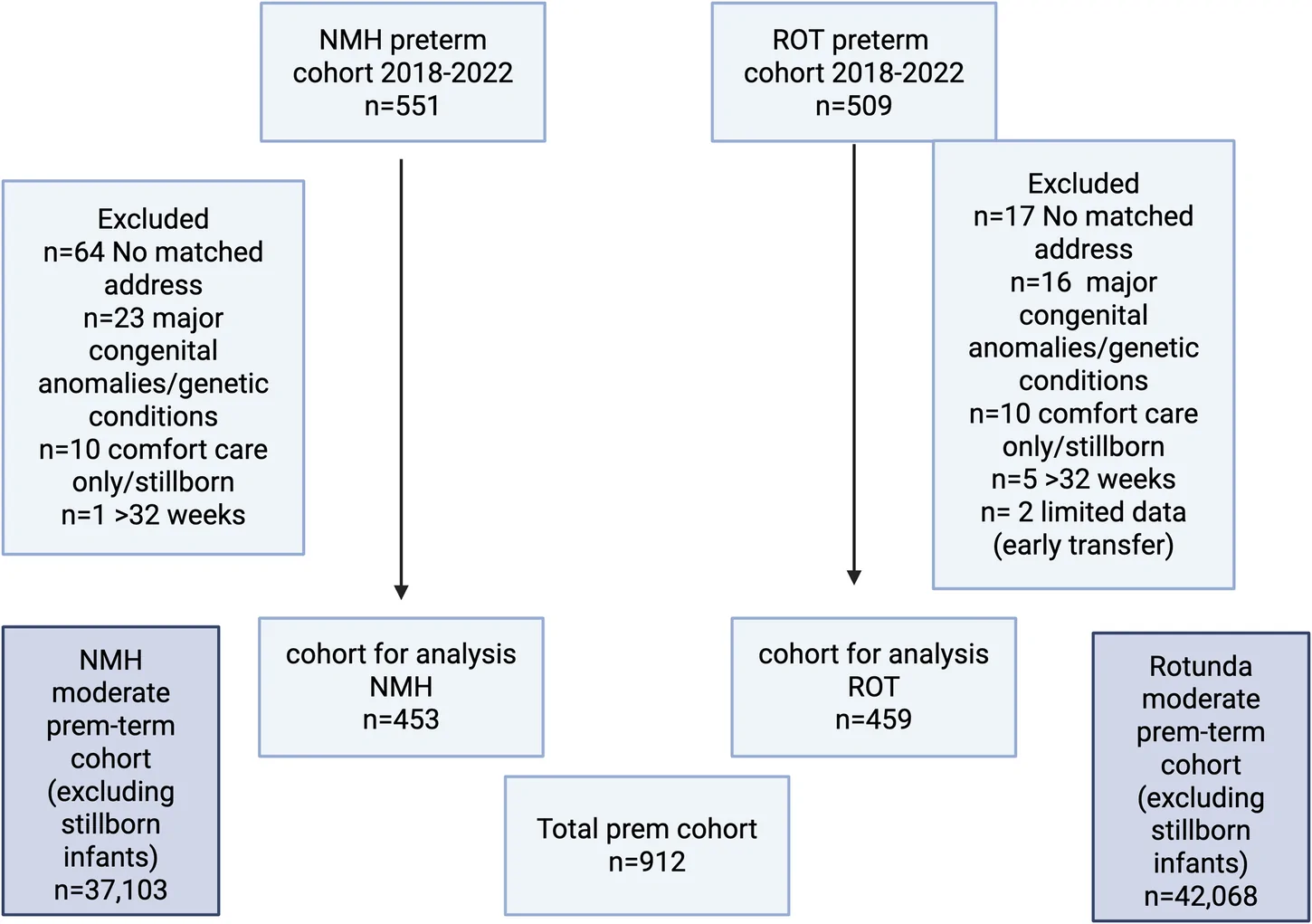
Flow diagram demonstrating inclusion and exclusion of very preterm neonatal cohort.

Neonates born <32 weeks corrected gestational age were separately identified using the same system. Neonates born >32 weeks were included if they were liveborn and their recorded address on the system could be matched using GeoDirectory, the Irish postal database of commercial and residential properties, to a small area to generate the HP index for each patient. Neonates born <32 weeks were included if they could be matched to a small area using GeoDirectory, had no major congenital or genetic anomalies and received active resuscitation (Fig. 1). Consent was deemed unnecessary as data was derived retrospectively from routinely collected clinical data.

## Procedures

All neonates were matched to a small area where possible using GeoDirectory to generate the HP index. Small areas represented 50–200 household blocks within a geographic area. Data were collected by the authors (DOR, LM, SD and CMM) from patient records for infants <32 weeks. Data collected are displayed in Table 1 and include neonatal demographics, reason for delivery, complications of prematurity and clinical outcomes. The primary outcome was the risk of occurrence of very preterm birth ( < 32 weeks corrected gestational age), compared with later preterm or term birth, by HP index. Secondary outcomes included recognised complications of prematurity identified prior to discharge from the neonatal unit. Neonatal sepsis was defined by either the presence of a known pathogen on neonatal blood culture or the intention to treat for five days of antibiotics or more [13]. Bronchopulmonary dysplasia/chronic lung disease was defined by the Neonatal Research Network (NRN) definition of requirement for oxygen for 28 days or greater [14]. Necrotising enterocolitis (NEC) diagnosis was defined by intention to treat for 5 days or more medically (medical NEC) or the requirement for surgery (surgical NEC). Reason for preterm birth was as recorded in the mother’s clinical notes. Intraventricular haemorrhage (IVH) was reported by paediatric radiologists on each site. Death was defined as death prior to discharge from Neonatal Intensive Care Unit (NICU).

**Table 1:** Baseline characteristics of included neonates by site.

	Site 1	Site 2	Total
No. of neonates liveborn at <32 weeks corrected gestational age	459	453	912
Median gestational age (+/- IQR)	29 2/7 (26 5/7- 30 6/7)	29 (26 5/7-30 5/7)	29 1/7 (26 5/7-30 5/7)
No. per completed gestational age(week (%))			
• 23 weeks	21 (4·5%)	12 (2·7%)	33 (3·6%)
• 24 weeks	31 (6·7%)	26 (5·7%)	57 (6·25%)
• 25 weeks	37 (8·1%)	36 (7·9%)	73 (8%)
• 26 weeks	35 (7·6%)	52(11·5%)	87(9·5%)
• 27 weeks	40 (8·7%)	40 (8·8%)	80 (8·77%)
• 28 weeks	39 (8·5%)	57 (12·6%)	96 (10·5%)
• 29 weeks	65 (14·2%)	59 (13%)	124 (13·5%)
• 30 weeks	80 (17·4%)	78 (17·2%)	158 (17·3%)
• 31 weeks	111 (24·2%)	93 (20·5%)	204 (22·4%)
Gender (No. of male, %)	231 (50·3%)	257 (56·8%)	488 (53·5%)
Birth weight (grams)	1195 (827-1505)	1175 (830-1525)	1185 (829-1520)
Maternal age	33 (29-37)	34 (30-37)	33 (29-37)
Multiple birth			
• Twins	118 (25·7%)	122(26·9%)	240(26·3%)
• Triplets	12 (2·6%)	29 (6·4%)	41 (4·5%)
• Quadruplets	4 (0·9%)	0 (0)	4 (0·4%)
Reason for delivery	Spontaneous Preterm Labour- 178 (38·8%) Preeclampsia-82 (17·9%) Antepartum haemorrhage -40 (8·7%) Clinical chorioamnionitis-33 (7·2%) NRCTG-22 (4·8%) Maternal reasons-22 (4·8%) IUGR-15 (3·3%) AEDF-12 (2·6%) TTTS-9 (2%) Cord prolapse-5 (1%) Other cause- 41 (8·9%)	Spontaneous preterm labour-209 (46·1%) Preeclampsia-52 (11·5%) Antepartum haemorrhage -36 (7·9%) Clinical Chorioamnionitis-39 (8·6%) NRCTG-37 (8·16%) Maternal reasons-8 (1·8%) IUGR-5 (1·1%) AEDF-37 (8·16%) TTTS-23 (5%) Cord Prolapse-2 (0·4%) Other cause-5 (1·1%)	Spontaneous preterm labour-387 (42·4%) Preeclampsia-134 (14·7%) Antepartum haemorrhage -76 (8·3%) Clinical Chorioamnionitis-72 (7·9%) NRCTG-59 (6·5%) Maternal reasons-30 (3·3%) IUGR-20(2·2%) AEDF-49 (5·4%) TTTS-32 (3·5%) Cord Prolapse-7 (0·8%) Other cause-46 (5%)
Rupture of membranes? (%)	209 (45·5%)	230 (50·7%)	439 (48·1%)
Average duration of rupture of membranes(hours)	48 (9-240)	56 (5·78-215)	56 (7·63-236)
Antenatal Steroids			
None	38 (8·3%)	20 (4.4%)	58 (6·4%)
Partial	83 (18%)	77 (17%)	160 (17·5%)
Complete	338(73·6%)	356 (78·6%)	694 (76·1%)
MgSO4	384 (83·7%)	381 (84·1%)	765 (83·9%)
CRIB II score	6(3-10)	7 (4-10)	7 (4-10)
Apgar score			
1 min	6 (5-8)	6 (5-8)	6 (5-8)
5 min	8 (7-9)	8 (7-9)	8(7-9)
Any mechanical ventilation	262 (57%)	216 (47·6%)	478(52·4%)
Days on mechanical ventilation	3 (1-10)	3 (1-7)	3 (1-8)
Days on non-invasive ventilation	13 (6-33)	14 (4-39)	13 (5-36)
Bronchopulmonary Dysplasia (%)	183 (39·9%)	186 (41%)	369 (40.4%)
Diagnosis of Sepsis	116 (25·2%)	104 (23%)	220 (24·1%)
Culture positive	65 (14·2%)	73(16·2%)	138 (15·13%)
Culture negative	51 (11·1%)	31 (6·8%)	82 (9%)
Number of neonates any antibiotics	419 (91·3%)	405 (89·4%)	824 (90·4%)
Days of antibiotic exposure	4·5 (2-10)	2 (1·5-6·5)	3 (1·5-7·5)
Diagnosis of Necrotising Enterocolitis	46 (10%)	47 (10·3%)	93 (10·2%)
Medical	31 (6·8%)	32 (7·0%)	63 (6·9%)
Surgical	15(3·2%)	20 (4·4%)	35 (3·8%)
Interventricular Haemorrhage	152 (33%)	107 (23·6%)	259 (28·3%)
Grades 1/2	114 (24·8%)	77 (17%)	191(20·9%)
Grades 3/4/PVL	38 (8·2%)	28 (6·2%)	66 (7·2%)
Death	48 (10·5%)	60 (13·25%)	108 (11·8%)

*IQR* Interquartile range.

## Statistics

To examine our primary outcome we compared the population distribution of area level socioeconomic status of neonates born ≥32 weeks to those born <32 weeks across both sites using the non-parametric two tailed Kolmogorov-Smirnov test. Secondary analysis examined the impact of the population distribution of area level socioeconomic status of neonates born ≥37 weeks to those born <32 weeks using the same method. To generate odds ratios to examine the impact of deprivation on very preterm birth, neonates were grouped into “Deprived” (HP index of -10 or less), “Average” (HP index from -10 to 10) and “Affluent” (HP index greater than 10) groupings as previously outlined, and a logistic regression performed [10]. Logistic regression was also used to examine the impact of HP index on complications of very preterm birth, with corrections for gestational age and birthweight. All statistics were performed using R statistical software [15].

### Role of the funding source

The funder of the study had no role in study design, data collection, data analysis, data interpretation, or writing of the report

## Results

**Fig. 2:**
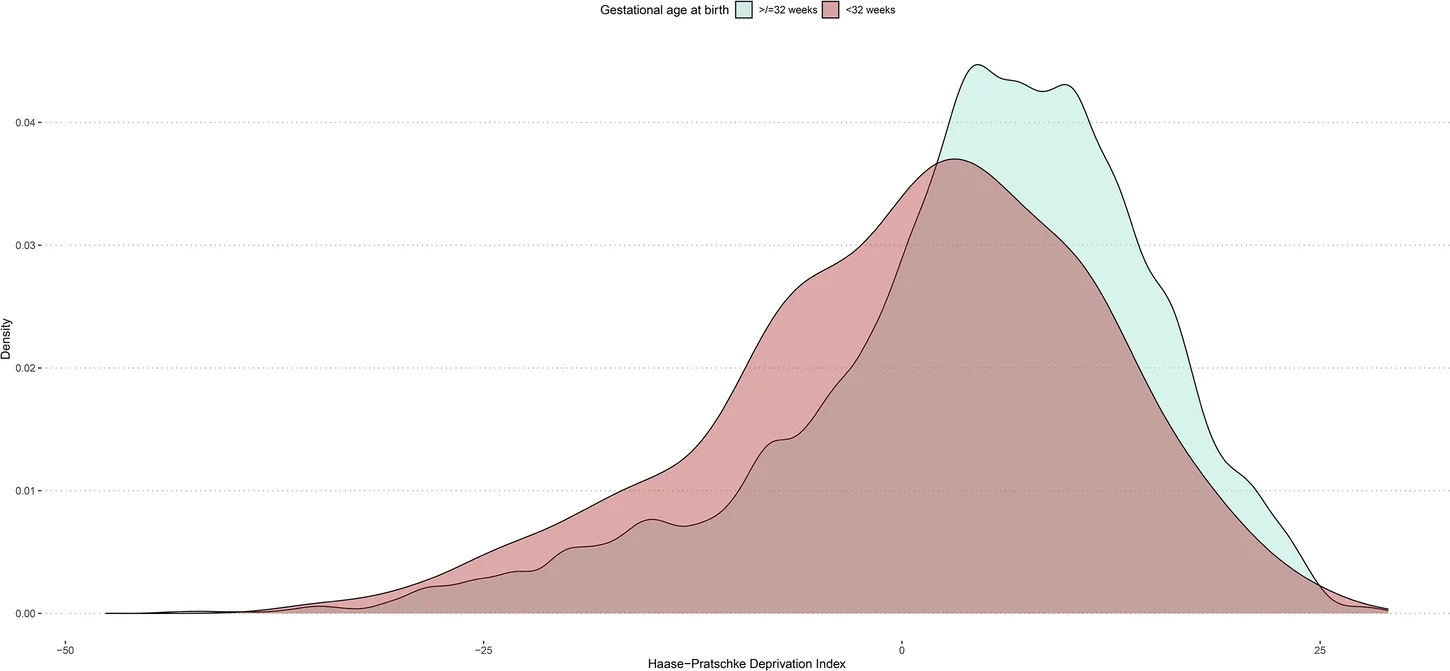
Density plot demonstrating different socioeconomic area scores of neonates born <32 weeks and those born at >/=32 weeks.

A total of 912 preterm neonates <32 weeks corrected gestational age were compared to the remainder of the population on both sites (*n* = 79,174). Characteristics of preterm neonates born on each individual site are presented in Table 1. To establish if the <32 week population was distinct from the >32 week population distribution groups were compared by Kolmogorov-Smirnov test using HP index as a continuous variable. This demonstrated a statistically significant difference between the HP index of neonates born <32 weeks and those >/=32 weeks of corrected gestational age (*p* < 0·001, Fig. 2). This finding was unchanged if the term population was limited to neonates >37 weeks (Supplemental Fig. 1). To establish the odds ratios of relative deprivation of preterm birth the analysed population were pooled into three groups to allow for comparison by logistic regression as described above. Baseline characteristics of the three groupings are described in Table 2. A clear effect of deprivation was demonstrated with neonates born into areas defined as deprived having an odds ratio of 1·5 (95%CI 1·20–1·74) for very preterm birth while those from affluent areas had an odds ratio of 0·61 (95% CI 0·51–0·72) compared to a reference group of neonates born in areas defined as average.

**Table 2:** Baseline characteristics of included neonates by socioeconomic deprivation cohort.

	Deprived	Average	Affluent
No. of neonates liveborn at <32 weeks corrected gestational age	146	576	190
Median gestational age (IQR)	29 (26 5/7-30 4/7)	28 3/7 (26 3/7-30 4/7)	30 1/7 (27 2/7-31)
No. per completed gestational age week (%)			
• 23 weeks	2 (1·36%)	27 (4·7%)	4 (2·1%)
• 24 weeks	13 (8·9%)	35 (6·1%)	9 (4·7%)
• 25 weeks	14 (9·6%)	46 (8%)	13 (6·8%)
• 26 weeks	11 (7·5%)	57 (9·9%)	19 (10%)
• 27 weeks	15 (10·3%)	51 (8·9%)	14 (7·4%)
• 28 weeks	17 (11·6%)	64 (11·1%)	15 (7·9%)
• 29 weeks	21 (14·4%)	85 (14·8%)	18 (9·5%)
• 30 weeks	27 (18·5%)	101 (17·5%)	30 (15·8%)
• 31 weeks	26 (17·8%)	110 (11·1%)	68 (35·8%)
Gender (No. of male, %)	77 (52·7%)	321 (55·7%)	90 (47·4%)
Birth weight (grams, IQR)	1180(832.5-1518)	1150(805-1480)	1335(947.5-1635)
Maternal age (IQR)	31 (26-35)	33(29-37)	34 (30-37)
Multiple birth (%)			
• Twins	36 (24·6%)	154 (26·7%)	50 (26·3%)
• Triplets	2 (1·3%)	27 (4·7%)	12 (6·3%)
• Quadruplets	0	4 (0·7%)	0
Reason for delivery (%)	Spontaneous preterm birth-64 (43·8%) Preeclampsia-25 (17·1%) APH-14 (9·6%) NRCTG-11 (7·5%) Clinical chorio-9 (6·2%) IUGR-2 (1·4%) TTTS-4 (2·7%) Maternal reasons-3 (2%) AEDF-9(6·2%) Cord prolapse-3 (2·1%) Other cause-2 (1·3%)	Spontaneous preterm birth-231 (40·1%) Preeclampsia-90 (15·6%) APH-47(8·2%) NRCTG-36 (6·25%) Clinical chorio-54(9·3%) IUGR-15 (2·6%) TTTS-18(3·1%) Maternal reasons-21 (3·6%) AEDF-28 (4·8%) Cord prolapse-2 (0·3%) Other cause-34 (5·9%)	Spontaneous preterm birth-92 (48·4%) Preeclampsia-19 (10%) APH-15(7·9%) NRCTG-12 (6·3%) Clinical chorio-9(4·7%) IUGR-3 (1·6%) TTTS-10 (5·3%) Maternal reasons-6 (3·2%) AEDF-12 (6·3%) Cord prolapse-2 (1%) Other cause-10 (5·2%)
Rupture of membranes? (%)	78 (53·4%)	273 (47·4%)	88(46·3%)
Average duration of rupture of membranes(hours (IQR))	67.83 (11.25-234)	56.5 (8.5-234)	45.22(3.48-192)
Antenatal Steroids (%)			
None	8 (5·5%)	40 (6·9%)	10 (5·2%)
Partial	27 (18·5%)	89 (15·5%)	44 (23·2%)
Complete	111 (76%)	447 (77·6%)	136 (71·6%)
MgSO4 (%)	118 (80·8%)	486 (84·4%)	161 (84·7%)
CRIB II score (IQR)	7(3-10)	7 (4-11)	5 (3-10)
Apgar score (IQR)			
1 min	6(4-8)	6(5-8)	7(5-8)
5 min	8(7-9)	8(7-9)	9(7-9)
Any mechanical ventilation (%)	77 (52·7%)	313 (54·3%)	88(46·3%)
Days on mechanical ventilation (IQR)	2 (1-6)	3(1-9)	3 (1-7)
Days on non-invasive ventilation (IQR)	13.5 (5-37)	15(5-36)	10 (4-32)
Bronchopulmonary Dysplasia (%)	57 (39%)	248(43·1%)	64 (33·6%)
Neonatal Sepsis (%)	36 (24·7%)	151 (26·2%)	33 (17·4%)
Culture positive	25 (17·1%)	93 (16·1%)	20 (10·5%)
Culture negative	11 (7·5%)	58 (10·1%)	13 (6·8%)
Number of neonates any antibiotics (%)	137 (93·8%)	519 (90·1%)	168 (88·4%)
Days of antibiotic exposure (IQR)	3 (1.5-7)	3(1.5-8)	1.5 (1.5-5)
Diagnosis of Necrotising enterocolitis (%)	16 (10·9%)	64 (11·1%)	13 (6·8%)
Medical	13 (8·9%)	41 (7·1%)	4 (2·1%)
Surgical	3 (2·1%)	23 (4%)	9(4·7%)
Intraventricular Haemorrage (%)	33 (22·6%)	181 (31·4%)	48 (25·3%)
Grades ½	23 (15·8%)	131 (22·7%)	39 (20·5%)
Grades 3/4/PVL	10 (6·8%)	50 (8·7%)	9 (4·7%)
Death (%)	15 (10·3%)	75 (13%)	18 (9·5%)

*IQR*Interquartile range

**Fig. 3:**
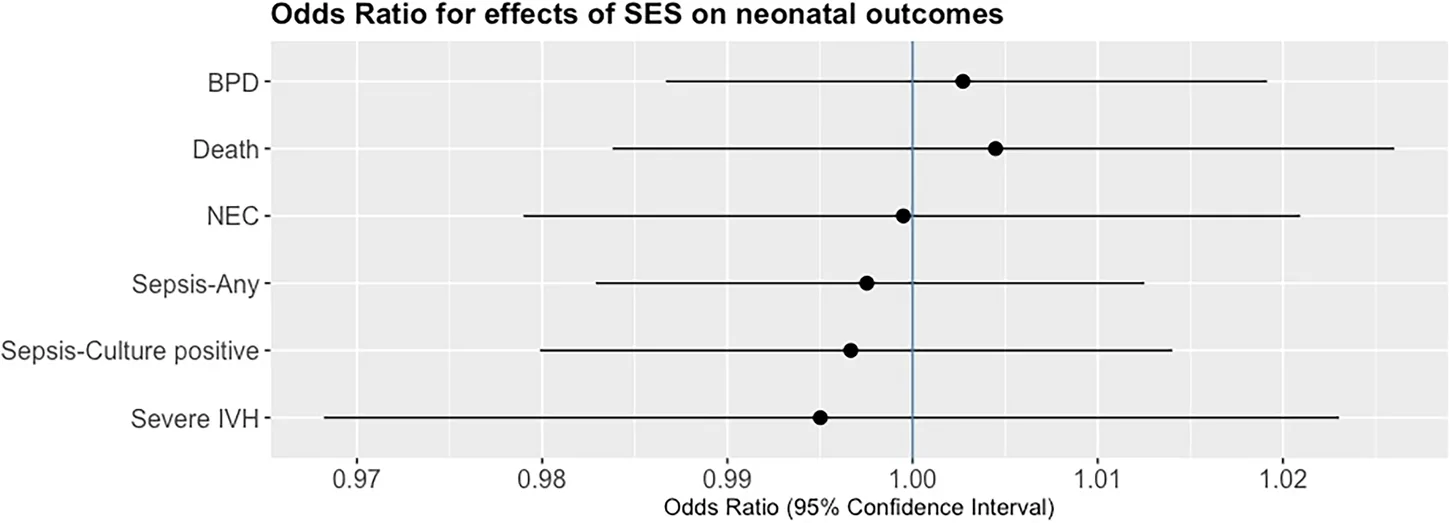
Forest plot demonstrating no statistically significant difference in key neonatal outcomes according to area level measure of socioeconomic deprivation.

To examine secondary outcomes of very preterm birth by socioeconomic status using HP index as a continuous variable, key neonatal comorbidities (BPD/CLD, IVH, NEC, Any sepsis, Culture proven sepsis) and death were analysed by logistic regression, correcting for both gestational age and birthweight. Socioeconomic status categories were not statistically compared given there was no statistically significant difference in the occurrence of any of these outcomes when analysed by socioeconomic status, as defined by HP index (Fig. 3). Additional modelling with additional confounders (multiple birth, maternal age) did not show a statistically significant difference (supplementary Table 1).

## Discussion

Deprivation has been associated with “any” preterm birth in other studies but there is limited data on neonates <32 weeks, who represent a smaller cohort than overall preterm births but carry a much greater burden of disease. This burden is not only in the acute outcomes examined in this research but also in longer term neurodevelopmental outcomes such as autism spectrum disorder, cerebral palsy and other developmental delays [16]. This study is the first to show, that families from socioeconomic deprived areas are at higher risk of very preterm birth in the Irish healthcare system.

Reassuringly while the odds of preterm birth were higher in parents from socioeconomically deprived areas, the odds of many of the acute outcomes of preterm birth of greatest concern were equivalent across the socioeconomic spectra, suggesting that care and short term outcomes of preterm neonates transcended any potential socioeconomic adverse effects once preterm delivery had occurred. Our cohort was confined to two large tertiary units who care for more than one hundred very preterm or very low birthweight neonates annually and correspondingly the occurrence of many outcomes, notably NEC and severe IVH were thankfully low. Although this was a large cohort of neonates the impact of socioeconomic status may have a modest effect size which was therefore not detected in this study.

There are limitations to our study: the overall proportion of babies with socioeconomic deprivation was low and the study was limited to two of the four national neonatal tertiary referral centres [17] accounting for 27% of total births. However, the hospitals chosen are representative of the country more generally as their location means that they provide care for differing levels of socioeconomic deprivation. Known confounders such as ethnicity and smoking were not available for much of the population studied. Although Ireland is a relatively ethnically homogenous population compared to other countries and has a relatively low smoking rate (reported at 6% in pregnant people) these factors are known to be subject to socioeconomic deprivation [18, 19]. Given the estimated effect of these factors on preterm birth in other studies, it is also unlikely that they would totally mitigate the effect size demonstrated in this study. Previous work examining mediators of socioeconomic inequalities in preterm birth showed variable impact with smoking most consistently demonstrating a mediating effect eliminating between 2 and 45% of observed effect sizes [20].

We also did not limit our study to singleton pregnancies as this would outrule a substantial percentage of our cohort and would be fundamentally unrepresentative of the very preterm population as a whole. Additionally, we could not identify which neonates from our >32 week cohort were multiple pregnancies due to limitations in how this data was generated. Importantly, properly taking the non-independence of multiple births would be more likely to lead to smaller standard errors and reduce the chances of a statistically significant result [21]. The null effect demonstrated in neonatal outcomes would therefore likely stand, albeit with more accurate variances.

Our findings support the fact that the risk of very preterm birth is associated with greater socioeconomic deprivation. This should guide health and public policy in supporting targeted antenatal care for vulnerable groups to attempt to mitigate the occurrence of very preterm birth in these areas as well as to focus postnatal support for neurodevelopmental premature infant aftercare.

## Supplementary information

Figure legend

Supplemental Figure

Supplementary Table

## Data Availability

An anonymised dataset will be shared with researchers on reasonable request and in compliance with GDPR by contacting the corresponding author.
