# Supplementary material for: Area level measures of socioeconomic deprivation and their impact on very preterm birth and very preterm birth outcomes: A retrospective cohort study
Source: J Perinatol. 2026 Apr 20;46(10):1972–8. doi: 10.1038/s41372-026-02674-1 (PMC13680268; doi:10.1038/s41372-026-02674-1)
Supplement: Supplementary file 1 [file 41372_2026_2674_MOESM2_ESM.pdf]

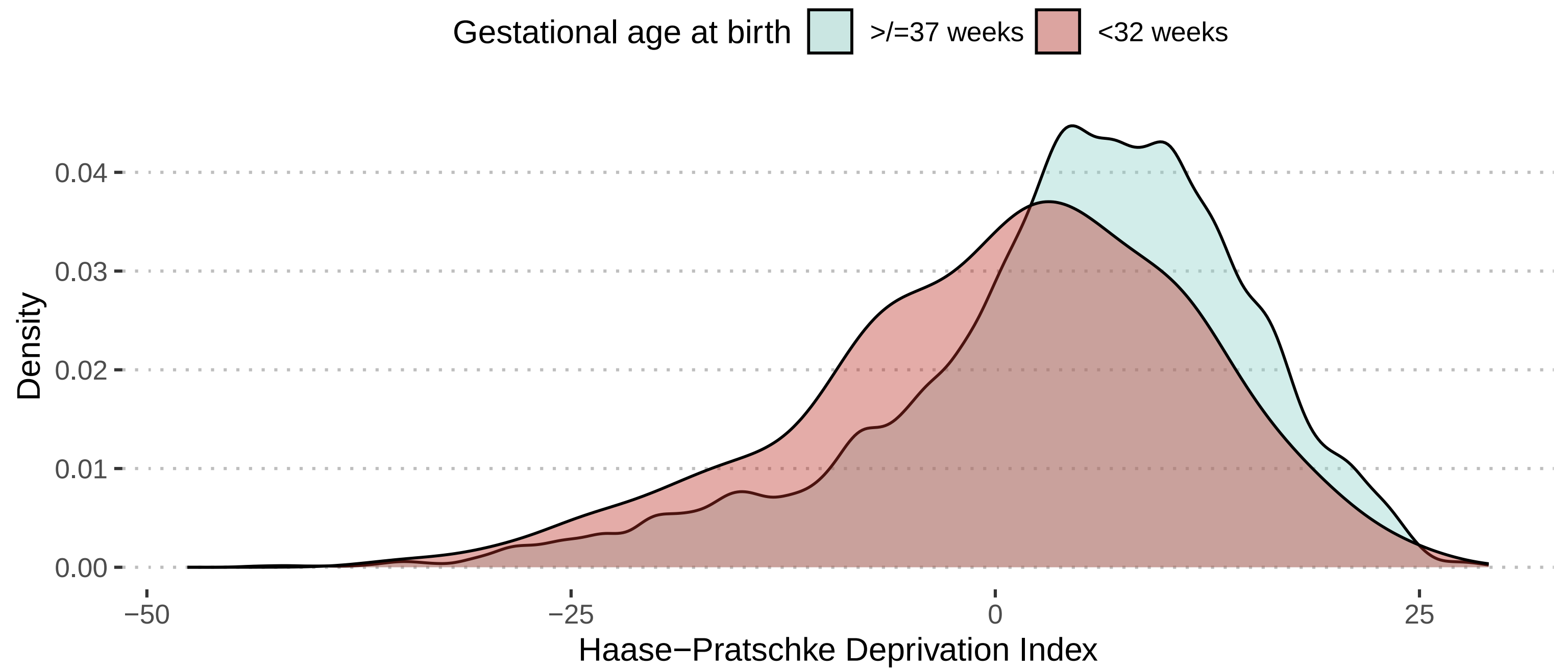
Gestational age at birth
>/=37 weeks
<32 weeks
Density
0.04
0.03
0.02
0.01
0.00
−50
−25
0
25
Haase−Pratschke Deprivation Index
