# Supplementary material for: Area level measures of socioeconomic deprivation and their impact on very preterm birth and very preterm birth outcomes: A retrospective cohort study
Source: J Perinatol. 2026 Apr 20;46(10):1972–8. doi: 10.1038/s41372-026-02674-1 (PMC13680268; doi:10.1038/s41372-026-02674-1)
Supplement: Supplementary file 2 [file 41372_2026_2674_MOESM1_ESM.docx]

Supplemental figure: Demonstrating density plot comparing preterm births <32 weeks corrected gestational age versus births occurring over or on 37 weeks corrected gestational age by Haase Pratschke deprivation index.
