# Supplementary material for: Area level measures of socioeconomic deprivation and their impact on very preterm birth and very preterm birth outcomes: A retrospective cohort study
Source: J Perinatol. 2026 Apr 20;46(10):1972–8. doi: 10.1038/s41372-026-02674-1 (PMC13680268; doi:10.1038/s41372-026-02674-1)
Supplement: Supplementary file 3 [file 41372_2026_2674_MOESM3_ESM.docx]

Supplemental Table:Regression outcomes including maternal age, gestational age, birthweight and multiple birth as confounders

| Outcome | Odds Ratio | 95% CI Odds Ratio | p-value |
| --- | --- | --- | --- |
| Death | 1 | 0.98-1.02 | 0.556 |
| BPD | 1 | 0.98-1.02 | 0.674 |
| NEC | 1 | 0.97-1.03 | 0.944 |
| Any-Sepsis | 0.99 | 0.98-1.01 | 0.76 |
| Sepsis-Culture positive | 0.99 | 0.98-1.01 | 0.75 |
| Severe IVH | 0.99 | 0.96-1.02 | 0.58 |
